# Early post-surgical rehabilitation and functional outcomes of a traumatic ulnar nerve injury: a pediatric case report

**DOI:** 10.3389/fneur.2024.1351407

**Published:** 2024-02-07

**Authors:** Federica Fulceri, Chiara Marinelli, Giulia Ghelarducci, Anna Maria Nucci, Andrea Poggetti, Larisa Ryskalin, Marco Gesi

**Affiliations:** ^1^Department of Translational Research and New Technologies in Medicine and Surgery, University of Pisa, Pisa, Italy; ^2^Center for Rehabilitative Medicine “Sport and Anatomy”, University of Pisa, Pisa, Italy; ^3^Department of Pediatric Orthopedics and Traumatology, Azienda Ospedaliero Universitaria, Meyer Children Hospital, Florence, Italy; ^4^Hand and Reconstructive Microsurgery Unit, Azienda Ospedaliero-Universitaria Careggi, Florence, Italy

**Keywords:** peripheral nerve injury, ulnar nerve injury, ulnar nerve rehabilitation, functional rehabilitation, termino-terminal (end-to-end) neurorrhaphy, nerve repair

## Abstract

**Background:**

Peripheral nerve injuries (PNIs) of the upper limb are very common events within the pediatric population, especially following soft tissue trauma and bone fractures. Symptoms of brachial plexus nerve injuries can differ considerably depending on the site and severity of injury. Compared to median and radial nerves, the ulnar nerve (UN) is the most frequently and severely injured nerve of the upper extremity. Indeed, due to its peculiar anatomical path, the UN is known to be particularly vulnerable to traumatic injuries, which result in pain and substantial motor and sensory disabilities of the forearm and hand. Therefore, timely and appropriate postoperative management of UN lesions is crucial to avoid permanent sensorymotor deficits and claw hand deformities leading to lifelong impairments. Nevertheless, the literature regarding the rehabilitation following PNIs is limited and lacks clear evidence regarding a solid treatment algorithm for the management of UN lesions that ensures full functional recovery.

**Case presentation:**

The patient is a 11-year-old child who experienced left-hand pain, stiffness, and disability secondary to a domestic accident. The traumatic UN lesion occurred about 8 cm proximal to Guyon’s canal and it was surgically treated with termino-terminal (end-to-end) neurorrhaphy. One month after surgery, the patient underwent multimodal rehabilitative protocol and both subjective and functional measurements were recorded at baseline (T0) and at 3- (T1) and 5-month (T2) follow-up. At the end of the rehabilitation protocol, the patient achieved substantial reduction in pain and improvement in quality of life. Of considerable interest, the patient regained a complete functional recovery with satisfactory handgrip and pinch functions in addition with a decrease of disability in activities of daily living.

**Conclusion:**

A timely and intensive rehabilitative intervention done by qualified hand therapist with previous training in the rehabilitation of upper limb neuromuscular disorders is pivotal to achieve a stable and optimal functional recovery of the hand, while preventing the onset of deformities, in patients with peripheral nerve injuries of the upper limb.

## 1 Introduction

Peripheral nerve injuries (PNIs) of the upper limb caused by direct trauma of the arm or secondary to fractures are very common conditions and may result in considerable disability depending on the severity and nerve involved (1). One of the major problems in surgery and post-operative management of upper limb PNIs is their unpredictable final outcome (2).

However, it is worth of mentioning that ultrasound (US) assessment of the peripheral nerves is increasingly becoming a valuable diagnostic tool to support the physical and electrophysiological examinations. This is particularly meaningful for patients with post-traumatic injury of the peripheral nerves where US imaging in the acute phase can promptly provide crucial data for the neurosurgeon to plan a tailored surgical approach (3).

Among the nerves of the brachial plexus, the ulnar nerve (UN) is the most frequently and severely injured one, usually resulting in both sensory and motor deficits of the forearm and hand (4–7). This may be due to the fact that, unlike other nerves, UN is not protected by muscle or bone during its anatomical path, making it more prone to injury (8).

Clinical presentation of UN lesions often includes: (i) loss of thumb adduction strength; (ii) intrinsic muscle weakness leading to decreased pinch and grip strength; (iii) hypotrophy of the thenar eminence; (iv) hyperextension of metacarpo-phalangeal (MCP) joint of the thumb (Jeanne’s sign); (v) hyperflexion of interphalangeal (IP) joint of the thumb (Froment’s sign); (vi) inability to maintain MCP joint in flexion; (vii) clawed-like appearance of the 4th and 5th digits, with typical hyperextension at the MCP joints and flexion of the proximal and distal IP joints; (viii) flattening of the palmar arch of the hand; (ix) loss of opposition of the 5th digit (9). Moreover, sensory complaints may include the loss of the ulnar border of the hand (from the 5th digit and downward) and of the haptic feedback for the integration of hand posture with precision activities performed by the first three digits (10).

In contrast to radial or median nerve lesions, UN injuries show a lower chance of functional recovery (9, 11–14). Even if an immediate repair of the UN is carried out, unfortunately in most cases, patients still present ulnar claw hand deformity and residual hand disability during recovery (15). This, in turn, is especially meaningful for the pediatric population, who may potentially suffer for decades (16). Indeed, an inadequate management of UN injuries can result in severe hand deformity that negatively impacts a child’s participation in activities of daily living (ADLs) with subsequent high psychological burdens. Conversely, timely and accurate rehabilitation is crucial to optimize the recovery of sensorimotor functions and outcomes following UN lesions. Even though most reports indicate that the prognosis for PNIs is better in children than adults (17–19), however the compliance of younger patient is usually poor (19). Indeed, pain, anxiety and stress can hinder the execution of functional testing manoeuvers as well as mask or exacerbate the symptoms.

At the same time, at present, a clear rehabilitation process for the treatment of brachial plexus injuries has not been developed yet (20). Despite much knowledge exists on the mechanisms of nerve injury and regeneration, the current literature lacks guidance and solid treatment algorithms for the postoperative management of UN injuries especially within the pediatric population (10, 19, 21), which instead could be crucial to preserve intrinsic motor function, while preventing hand deformities or sensory abnormalities.

This present case study describes a 11-year-old child who experienced left-hand pain, stiffness, and disability secondary to a traumatic UN lesion, who received nerve repair immediately. Then the patient underwent an early rehabilitation protocol consisting of multiple treatment techniques and physical modalities. Follow-up examinations were conducted at 3 and 5 months after nerve repair and focused on three main points: sensory and motor recovery, and quality of life evaluated as time of return to daily activities. Early and multimodal hand rehabilitation has been shown to be beneficial in improving overall patient’s condition, resulting in favorable outcomes. In fact, at the end of the rehabilitation protocol, the patient achieved substantial improvement in pain and quality of life scores. Of considerable interest, the patient achieved a complete functional recovery along with satisfactory improvements of dexterity and power grip functions.

## 2 Case presentation

**Figure 1:**
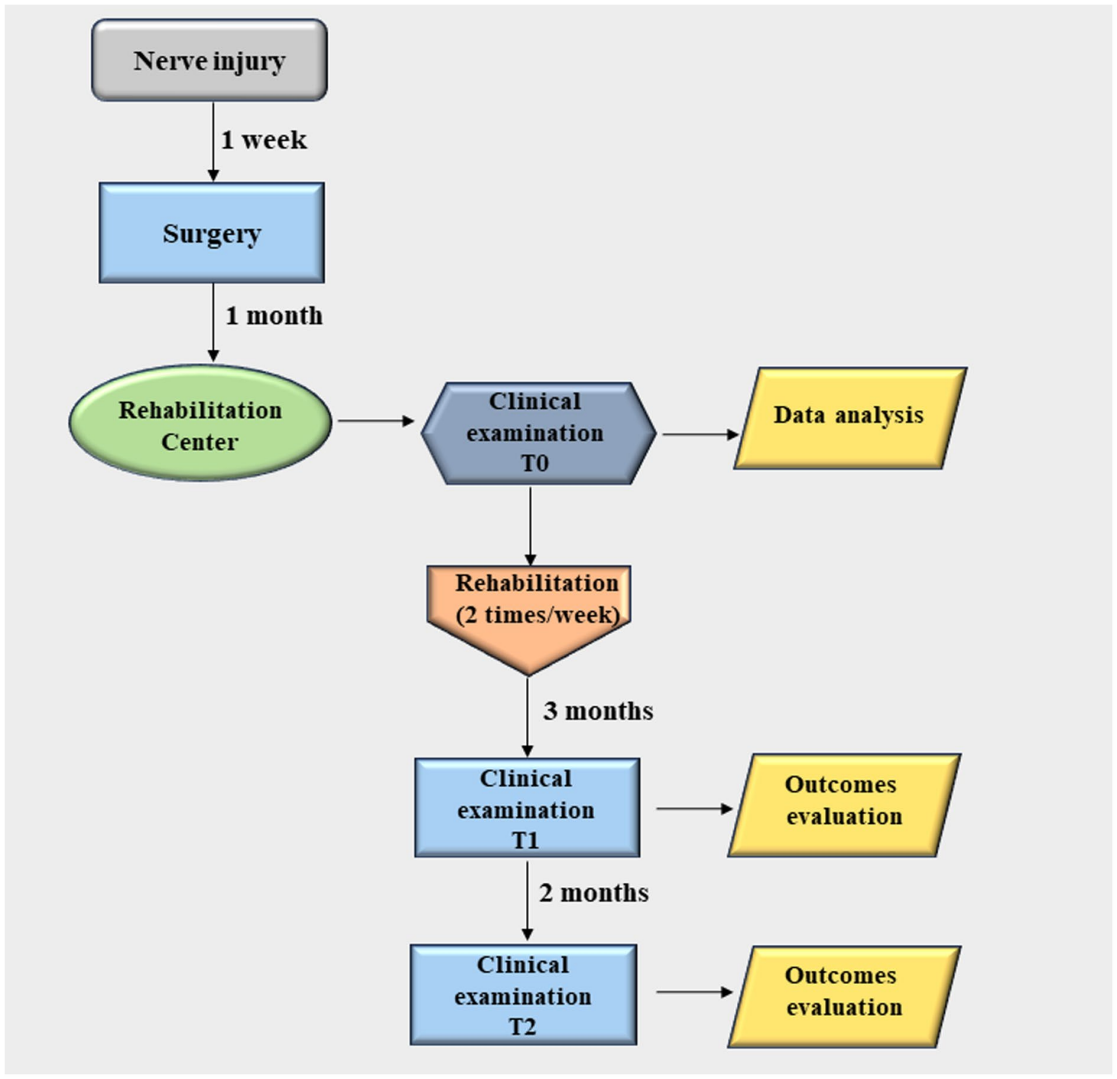
Flow chart of the study.

**Figure 2:**
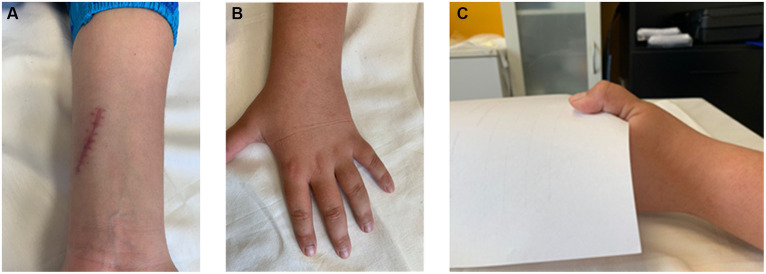
Clinical presentation of the ulnar nerve injury. **(A)** Representative picture demonstrating the surgical incision in close proximity to the Guyon’s canal; **(B)** Flattening of the palmar arch of the hand due to a reduction of MCP joint flexion; **(C)** The occurrence of a positive Froment’s sign is evident as a bending of the distal tip of the thumb when attempting to pinch a piece of paper between the thumb and the index finger.

We report a case of a 11-year-old child who approached the Centre for Rehabilitative Medicine “Sport and Anatomy” of the University of Pisa for UN palsy due to acute nerve transection at his left hand, following a domestic accident (Figure 1). The nerve lesion, secondary to a clear-cut injury (i.e., knife wound) to the wrist, occurred upstream of Guyon’s canal (Figure 2A), and resulted in important motor and sensory disabilities, due to the involvement of both sensory and motor branches. The nerve lesion was treated surgically with termino-terminal (end-to-end) neurorrhaphy at the Department of Pediatric Orthopedics and Traumatology, Azienda Ospedaliero Universitaria, Meyer Children Hospital, Florence, Italy. The patient came to our observation approximately 1 month after surgery. Upon initial examination, the patient presented a classic claw hand deformity, a hypotrophy of interossei and lumbrical muscles, progressive retraction, joint stiffness, and flattening of the transverse metacarpal arch (Figure 2B). The patient’s primary complaint was intense pain, along with stiffness and hand musculature weakness. Alongside with hand clawing, an increased hyperextension of the MCP joint and flexion of IP joints were also observed. Remarkably, the patient showed difficulty in MCP joint flexion and IP joint extension at the level of the 4^th^ and 5^th^ fingers. Indeed, although still possible, this activity was somewhat weaker than normal functional. Furthermore, the imbalance between interossei and lumbrical muscle groups and extensor digitorum communis (EDC) and extensor digiti minimi (EDM, also known as extensor digiti quinti proprius) caused MCP joint hyperextension, which was maintained over time. The paralysis of the abductor pollicis brevis (APB), flexor pollicis brevis (FPB), and first dorsal interosseous (1DI), along with the loss of opponens pollicis (OP) muscle strength resulted in a severe hypotrophy of the thenar eminence. Finally, a diminished pinch strength of the thumb (e.g., pulling on a piece of paper) was observed due to the paralysis of the adductor pollicis (AP), of the deep head of FPB, and of the 1DI. Again, a hyperextension (10°-15°) MCP joint of the thumb was evident (Jeanne’s sign) along with the hyperflexion of the IP joint (Froment’s sign) (22) (Figure 2C).

**Figure 3:**
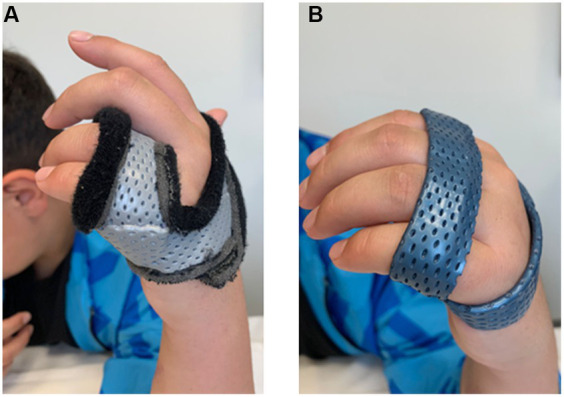
Examples of the two splints for ulnar nerve injury. Pictures of the two static splints designed to prevent hand clawing **(A)** and MCP joints hyperextension **(B)**, respectively.

The rehabilitative protocol started 1-month post-surgery, two sessions per week, for five successive months. In detail, we developed a multimodal treatment plan which was tailored to patient’s specific needs and goals for recovery with the aim of obtaining an effective rehabilitation in the shortest amount of time possible. This latter consisted of multiple treatment techniques and physical modalities, each one with clear goals, and included paraffin thermal therapy, strengthening and joint mobilization programs, scar management, pain desensitization techniques, transcutaneous electrical nerve stimulation (TENS), and splinting (Supplementary Table 1). Remarkably, paraffin wax bath combined with exercise improved mobility, decrease stiffness, and increase tissue elasticity of the post-traumatic stiff and painful hand (23, 24). Pain management approaches comprised pain desensitization techniques and TENS. Wound scar management was also performed to reduce joint stiffness–tissue adherence (i.e., the tension forces that can cross the scar) and pain, while obtaining a slow stretching through static posture and compression. Furthermore, musculature strengthening exercises were performed to recover muscle wasting of the thenar eminence, interossei, and lumbrical muscles. Finally, two custom-made splint orthoses of thermo-modeling materials were provided (Figure 3).

In detail, the first splint was positioned at MCP joints of 4th and 5th fingers to keep them at 70° in a flexed position thus preventing their clawing (Figure 3A). The patient was instructed to wear the static splint throughout the day for all activities of daily living. The second one was designed for all MCP joints and positioned at 45° to prevent hyperextension while allowing active flexion and supporting the transverse arch of the hand (Figure 3B). This latter splint had to be alternated with the first during daily activities, while it had to be worn continuously throughout the night.

The patient was treated for 5 months, and the following subjective and functional outcomes were considered:

- Reported pain severity, assessed using the Numeric Rating Scale (NRS), where scores ranged from 0 (no pain) to 10 (most severe pain). The NRS scale is a unidimensional and reliable measure of pain intensity that is widely used in clinical practice (25).
- Power grip, measured with a Hydraulic Handgrip Dynamometer (North Coast Medical, CA, USA), which estimates muscle strength primarily generated by intrinsic (Jamar I) and extrinsic (Jamar II) flexor muscles strength (26). The handgrip force is displayed on the dynamometer in kilograms (with a maximum of 90 kg). The patient is encouraged to produce maximal grip strength in one go for at least 5 s and relax thereafter. The test was repeated three times with a 20-s rest interval, and then the average of power grip was calculated.
- Precision grip, measured with a hydraulic Pinch Gauge (North Coast Medical, CA, United States) (27). The patient was asked to seat in a standardized position with shoulder adducted, elbow flexed at 90°, forearm, and wrist in a neutral position, and to press as firmly as possible (28). Maximum pain-free pinch strength was measured with respect to the standard key-pinch, using a single measurement (29). The test was repeated three times with a 20-s rest interval, and then the average of precision grip was calculated.
- Hand disability, evaluated with the Quick-Disabilities of the Arm, Shoulder, and Hand (QuickDASH) questionnaire. This latter asks about the symptoms as well as the patient’s ability to perform certain activities, and it scored from 0 (best function, no limitation) to 100 (worst function, full disability). The QuickDASH is a shortened version of the self-administered DASH questionnaire, and it contains only 11 items instead of the 30-item DASH outcome measure (30).

Statistical analysis was performed with StatView software. Data obtained from Jamar I, Jamar II and Pinch tests at T0, T1 and T2 time points were compared using the analysis of variance, Anova, followed by Scheffe’s *post hoc* test. Data were expressed as mean ± standard error. The null hypothesis (H_0_) was rejected when *p* < 0.05.

Under prior informed consent, three evaluations were performed, namely at the beginning of the treatment (T0), at 3-month follow-up (T1), and at the end (T2) of the rehabilitation program (i.e., 5 months after the beginning of the rehabilitation protocol). Overall, the rehabilitative program was generally well tolerated by the patient, who did not report any complications associated with the treatment received.

Primary outcome was a decrease in self-reported pain using the NRS scale. As reported in Table 1, referred pain intensity showed a linear decrease across the intervention, being more than halved at the end of the rehabilitative protocol.

**Table 1:** Assessment of the motor function recovery.

Timepoint	NRS	Jamar I	Jamar II	Pinch	QuickDASH
T0	7	4 ± 0	6 ± 0	1.67 ± 0.17	77.30%
T1	5	6 ± 0^*^	8 ± 0	2.17 ± 0.17	34.10%
T2	3	7.17 ± 0.60^*^	9.67 ± 1.20^*^	2.50 ± 0.29	25%

Outcomes at baseline (T0) and after 3 (T1) and 5 months (T2). ^*^p < 0.05 vs. T0.

Secondary outcomes included handgrip and pinch strength. Compared to first-day evaluations (T0), there was a significant increase in both intrinsic (Jamar I) and extrinsic (Jamar II) flexor muscles strength at the end of the rehabilitation protocol (T2) (Table 1). Of note, Jamar I and Jamar II were restored up to 71.70% and 76.32% of the contralateral (uninvolved) hand, respectively. Again, a noticeable improvement in the key pinch strength of the thumb was recorded, corresponding to the 45.45% of the right hand at the end of the rehabilitation protocol.

Finally, with reference to hand disability, the patient had greatly improved functional outcomes at 5-month follow-up. Indeed, the QuickDASH score decreased from 77.3% at T0 up to 25% at T2. Remarkably, this latter score corresponded to a minimal disability which did not significantly impact on ADL performance. Of note, for the QuickDASH, a change of 20 points was identified as the most adequate values to determine the minimal clinically important difference (MCID) in patients with upper-limb musculoskeletal disorders (31).

## 3 Discussion

Because of its peculiar anatomical path, traumatic injuries of the UN are common and often result in unsatisfactory and incomplete sensory-motor recovery. UN injuries usually present with a complaint of intense pain, sensory abnormalities, and claw hand deformity having a severe impact on the performance of simpler daily tasks. This, in turn, negatively impacts the patient’s quality of life and functional independence. Within this frame, patient adherence to prescribed exercises and/or orthosis wear is meaningful to predict positive functional outcome and avoiding hand deformities. This is particularly challenging for the therapists working with pediatric patients, where poor compliance is commonly encountered. As a consequence, pediatric patients may experience reduced treatment outcome and lifelong impairments.

Besides the time elapsed between nerve injury and surgery and the surgical method applied, the effectiveness of a physical therapy rehabilitation for UN injury largely depends on the adopted physical rehabilitation program. However, among potential factors influencing patient’s compliance, the fear of the pain and/or the relatively poor tolerance to pain are one of the most significant barriers to therapy adherence among young patients. This is mainly because an increase in perceived pain may result in physical discomfort and emotional state of tension which further counter for adherence to treatment. In contrast, physical therapies and/or exercises that relieved pain and/or other symptoms appears to be strong facilitators to boost physiotherapy adherence (32, 33).

Therefore, in the first phase of our rehabilitation protocol, the primary outcome was to decrease pain symptoms, in order to restore proper functionality and reduce hand disability. Remarkably, a significant reduction was observed in pain intensity across the intervention following the use of paraffin thermal therapy, which enabled a broad array of therapeutic implications including hyperemia, myorelaxation, and anti-edema action. Furthermore, exercise after wax bath is essential to increase muscle circulation. Again, the sedative effect of heat can result in increased muscle strength and range of movement. This is in line with the evidence showing that paraffin wax bath treatment followed by joint mobilization techniques are more effective and result in greater improvements of range of motion than mobilization alone in the rehabilitation of the stiff hand (23, 34). Moreover, paraffin wax has shown to be useful for stretching scars and adhesions, making the skin and soft tissues softer, moister, and pliable (35). This, in turn, improves patient compliance and allowed subsequent mobilization work, aimed at recovering muscle wasting and maintaining range of motion and tissue elasticity. At the same time, the reduction in pain symptoms allowed to intensify sensory re-education through desensitization techniques. At present, there is still not a single technique that ensures the full recovery of tactile discrimination of the hand of an adult after a peripheral nerve injury (36, 37). Therefore, paraffin thermal therapy may be useful to enhance the beneficial effects of sensory re-education strategies after repair.

In the last rehabilitation phases, both active and global strengthening exercises were carried out to increase muscle tone and trophism, which resulted in a noticeable linear increase in handgrip and pinch strength. Of note, the muscle strengthening program allowed us to increase muscle tone and trophism, especially of the lumbrical, dorsal and palmar interossei muscles, and restoring agonist/antagonist muscle balance (Supplementary Figure 1). Furthermore, our rehabilitation protocol reduced muscular asthenia and improved patient’s autonomy in performing ADLs.

Compared to data reported in the literature (2, 9, 38), which indicates that a stable and optimal functional recovery can be expected at an average of 30 weeks following isolated ulnar nerve injuries, we achieved better results with reduced timing.

This may be due to the use of a multimodal rehabilitative approach which combined paraffin thermal therapy, active hand mobilization, and custom-made splint orthoses. These latter where pivotal in preventing joint stiffness and muscle contractions. Indeed, it is reported that proper splinting encourages early use on injured hand during ADLs while minimizing the occurrence of deformities (39). In contrast, prolonged muscle imbalance can cause joint contracture and muscle over-stretching which may limit hand function recovery.

Although the patient still reported a very slight difficulty due to the presence of paresthesia, at the end of our rehabilitative program, the complete functional recovery of the affected hand was accompanied by a lesser disability and a substantial improvement in ADLs performance. This is in line with current literature showing that a better recovery of motor function of the hand positively affects patient’s quality of life (2, 17).

Acknowledging the limitation of reporting a case report, it can be said that our tailored and specialized rehabilitative intervention resulted in remarkable and successful clinical results which support the validity of our proposed protocol for the management of UN injuries. The current results showed that a timely and multimodal rehabilitative treatment is pivotal to regain a full functional recovery while avoiding the onset of limb deformities.

## 4 Conclusion

The purpose of this report is to bring attention to UN injury management, with a particular regard to those occurring within pediatric population. In fact, even after an attempted repair, functional outcomes are difficult to achieve, and normal hand function is seldom restored. At present, a clear consensus regarding the correct timing, progression of exercises and TENS protocol, and appropriate treatment approaches for the management of UN injuries is lacking. Thus, finely tuned research regarding reproducible and solid rehabilitative treatment algorithms for this condition is urgently needed.

## Data availability statement

The raw data supporting the conclusions of this article will be made available by the authors, without undue reservation.

## Ethics statement

Ethical review and approval were waived for this treatment since it was part of ordinary clinical activity. The studies were conducted in accordance with the local legislation and institutional requirements. Written informed consent for participation in this study was provided by the participants’ legal guardians/next of kin. Written informed consent was obtained from the minor (s)’ legal guardian/next of kin for the publication of any potentially identifiable images or data included in this article.

## Author contributions

FF: Conceptualization, Data curation, Formal analysis, Writing – original draft, Writing – review & editing. CM: Data curation, Methodology, Writing – review & editing. GG: Data curation, Methodology, Writing – review & editing. AMN: Methodology, Writing – review & editing. AP: Methodology, Writing – review & editing. LR: Conceptualization, Data curation, Formal analysis, Writing – original draft, Writing – review & editing. MG: Conceptualization, Funding acquisition, Methodology, Supervision, Writing – review & editing.
