# Supplementary material for: Early post-surgical rehabilitation and functional outcomes of a traumatic ulnar nerve injury: a pediatric case report
Source: Front Neurol. 2024 Feb 7;15:1351407. doi: 10.3389/fneur.2024.1351407 (PMC10879349; doi:10.3389/fneur.2024.1351407)
Supplement: Supplementary file 1 [file Table_1.DOCX]

Supplementary Material

**Supplementary Table 1.** Multimodal rehabilitation protocol for ulnar nerve injury.

| **REHABILITATION TECHNIQUES** | **TIMING** | **FREQUENCY** | **PROCEDURE** | **BENEFITS** |
| --- | --- | --- | --- | --- |
| Paraffin wax | 15 min | 2 times *per* week | Spread the fingers and dip the hand into the wax (at least 3-4 times); remove the hand once coated and cover it with a plastic bag; then, carefully peel off the cooled wax from the hand. | Reduce pain and edema.  Improve tissues elasticity. |
| Hand mobilization | 15 min | 2 times *per* week | Pompage technique: traction, maintenance, and relaxation of the hand joints. | Improve joint lubrication and fluidity of movements. |
| Active hand exercises | 15 min | 2 times *per* week | Activation of intrinsic and extrinsic hand muscles. | Reinforce deficient muscles after the trauma |
| Desensitization techniques | 10 min | 1 time *per* week | Rub the fingers with a variety of textures following a pyramid of sensitivity (from the most pleasant texture to the most irritating one) | Reactivate and rebalance mechanoreceptors of the hyperesthesic scar area. |
| Scar management | 10 min | 2 times *per* week | Manual rolling and pinching of tissue adhesions. | Improve movement by an adequate tissue sliding. |
| Electric muscle stimulation technique | 20 min | 2 times *per* week | Exponential (triangular) pulse for activation of denervated muscle fibers. | Maintain muscle trophism in the absence of voluntary contraction. |


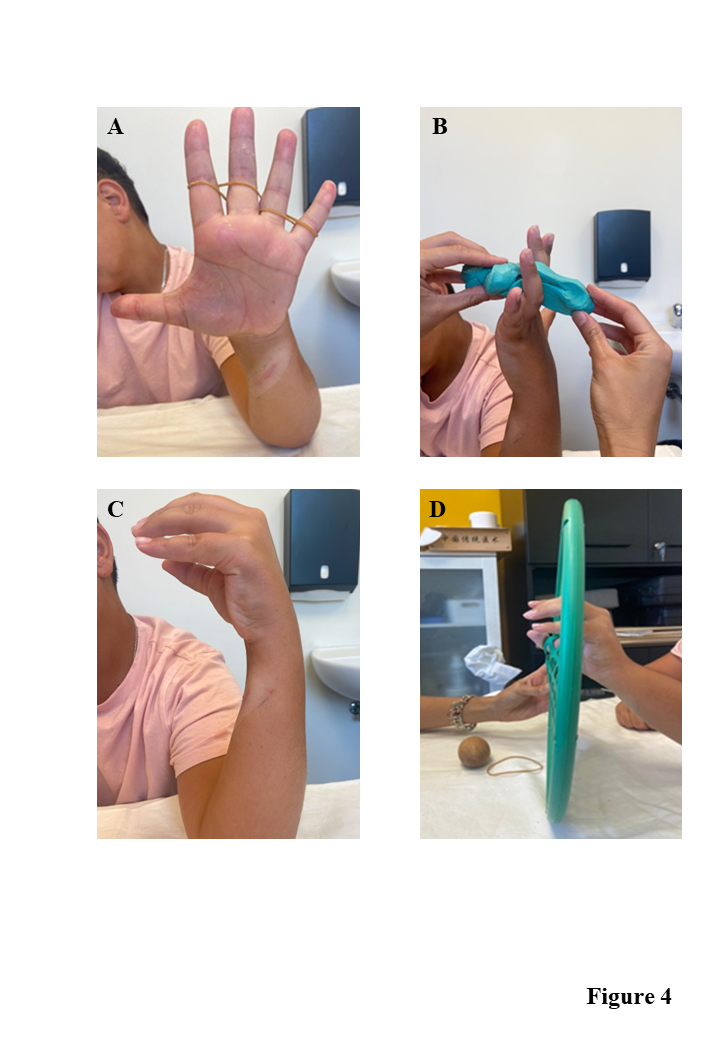


**Supplementary Figure 1. Representative pictures of hand strengthening exercises.** (A) Exercise for strengthening the dorsal interossei muscles is performed by placing a rubber band around the fingers and slowly pushing (abducting) the fingers against the band. (B) Palmar interossei muscles strengthening exercise are performed by putting a silicon-based putty in between the fingers and squeezing the fingers all together in adduction. Some examples of lumbrical muscle strengthening exercise are reported in panels (C, D). In (C) the exercise is performed by forming a “rooftop” using straight fingers, leaving the thumb underneath; then, all fingers are pressed down toward the thumb, keeping fingers straight and together. In (D) a power-web exercise tool for hand strengthening is reported.
